# Quality of drug stores: Storage practices & Regulatory compliance in Karachi, Pakistan

**DOI:** 10.12669/pjms.325.9705

**Published:** 2016

**Authors:** Syed Shaukat Ali Muttaqi Shah, Baqar Shyum Naqvi, Mashhad Fatima, Asif Khaliq, Abdul Latif Sheikh, Muhammad Baqar

**Affiliations:** 1 Mr. Syed Shaukat Ali Muttaqi Shah, M Pharm. PhD Scholar, Department of Pharmaceutics, Faculty of Pharmacy, University of Karachi, Karachi, Pakistan; 2 Dr. Baqar Shyum Naqvi; PhD. Professor, Department of Pharmaceutics, Faculty of Pharmacy, Hamdard University, Karachi, Pakistan; 3 Ms. Mashhad Fatima, MPhil. PhD Scholar, Department of Chemistry, University of Karachi, Karachi, Pakistan; 4 Mr. Asif Khaliq, MPH, MBA, Pharm D. Baqai Medical University, Karachi, Pakistan; 5 Mr. Abdul Latif Sheikh; MS(NY), R.Ph(NYS Board) Director, Pharmacy Services, Aga Khan University Hospital Karachi & Outreach Programme, Karachi, Pakistan; 6 Mr. Muhammad Baqar, Pharm D. Pharmacist, Medilink Clinics Pharmacy, Karachi, Pakistan

**Keywords:** Drug Stores, Regulatory Compliance, Storage Practices, Quality

## Abstract

**Objective:**

To assess and evaluate the drug storage quality and regulatory compliance among privately operated drug stores of Karachi Pakistan.

**Methods:**

A cross-sectional survey of drug stores located in Karachi was conducted from May to December 2013. A total of 1003 drug stores that were involved in the sales, purchase and dispensing of pharmaceutical products were approached by non-probability purposive sampling technique, and the information was collected using a close ended, structured questionnaire.

**Results:**

Out of 1003 drug stores inspected only 4.1%(n=41) were found compliant to regulatory requirements. Most of the stores 74.9%(n=752) were selling general items along with the drugs. Only 12%(n=124) stores were having qualified person working on the store, out of which 33% were pharmacist. 47.4%(n=400) of the stores had drug sales license displayed in the premises and 33.4%(n=282) of the stores had expired drug sales license. 11.4%(n=94) stores were found selling vaccines without proper refrigerator and only 11.7% stores had the power backup for the refrigerator. Only 40.2%(n=403) of stores were protected from direct sunlight and 5.4%(n=54) having air conditioning in the premises.

**Conclusion:**

The regulatory compliance of majority of the drug stores operated privately in different areas of Karachi is below standard. Only a few drugs stores have adequate facilities to protect the drugs from extreme temperature, sunlight and provision of refrigeration. Very few of the drug stores carry out drug sales under the supervision of qualified pharmacist. There is a dire need to improve the storage practices in the drug stores by complying with the regulatory standards/laws as specified by the Drug Regulatory Authority of Pakistan.

## INTRODUCTION

Drug storage and usage is one of the most important components in healthcare system. In many low-income countries, failure of government bodies to provide free healthcare, regulatory reforms and privatization have increased the unrestricted access of drugs to the general population through private pharmacies.^1^,^2^ These private pharmacies are often the first point of contact for patients seeking health care because of its proximity and being less socially distant compared to other providers, including medical doctors and traditional practitioners.^3^ Primarily activities carried out at the drug stores are buying and selling of drugs; most of the time over the counter and without a proper prescription. The sale of drugs in private stores has been associated with varying affordability, irrational use of drugs and availability of substandard and/or counterfeit drugs, which make the regulatory framework more challenging for developing countries.^1^,^2^

In low and middle income countries, the evidence related to the quality of professional community pharmacy services is limited, but data available refers to the deficiency of standards in the practice. Problems such as shortage of adequately trained human resource and lack of enforcement of regulatory structures are confronted by most of the developing countries, which results in free access of even prescription drugs to the patients.^4^,^5^ This unregulated sales of drugs is coupled with self-medication, being one of the prevalent phenomenon in developing countries, with varying percentages between 6.3-76% in different settings and demographic groups and is seen in both high and low income level populations. These factors makes medication usage more dangerous for the population and does warrants strict policy changes and implementation to curtail the issue.^6^-^9^

In Pakistan drug manufacturing companies don’t sell medications directly to the retail outlets on the contrary appoint distributors and/or wholesalers to supply drugs to the retailers from where it is dispensed to the patient. All these drug distribution facilities need to be licensed in the country. As per the Drug Act 1976, Government of Pakistan (GoP) has delegated regulation of: sale and specification of pre-conditions for sale of drugs to provincial government.^10^ The key requirements for the sale of drugs as described in the Sindh Drug Rules 1979 amended 27^th^ April 2010 are as follows: (i). Premises size must be > 100 square feet for retail and > 200 for pharmacy and wholesale, (ii) Proper facilities for drug storage for preserving drug quality and (iii) Drug Sales must be supervised by a registered pharmacist under Pharmacy Act 1967. Furthermore at every drug store the original valid drug sales license should be displayed prominently in the store.

There is no complete database of drug stores in the country, but roughly its estimated that there are around 45,000-50,000 retail drug and wholesale outlets in the country, out of which 15,000 stores are located in the province of Sindh.^11^ Only couple of small scale studies have been conducted previously in Pakistan to assess the storage and dispensing practices of drug stores in Pakistan, and the available data suggest the drug stores are operating below par with compromised storage conditions, with only 19.3% of the store meeting licensing requirements established by government authorities. Very few of the stores i.e. 12-22% were found to have staff with diploma or degree related to medical sciences or drugs.^11^-^13^

There is insufficient data on the operations of drugs stores in Pakistan, and none of the study explored the storage practices and regulatory compliance of drug stores in Karachi. This study is one of its kind to elaborate the storage practices and compliance of the drug stores to the established regulatory requirements. Karachi being the largest and most densely populated city and financial capital of Pakistan, is assumed to be well regulated compared to rural areas of Pakistan. The study would also serve as baseline for regulatory authorities to highlight the specific issues and formulate the focused action plan to enhance safe handling of drugs in the retail and wholesale drug stores in the region.

## METHODS

This is a descriptive cross-sectional study that was conducted from May 2013 to December 2013 among privately operated drug stores in Karachi, by non-probability purposive sampling technique. In order to keep consistency of information the drug stores which are solely dealing with herbal or other alternative medicine and the store operating inside government hospitals/clinics were excluded because of different nature of products and operations respectively.

To estimate the number of drug stores, list of drug stores served by two renowned pharmaceutical distributors operating in Karachi was obtained and combined. The lists were incomplete because these distributors were not supplying drugs to all the areas of Karachi. Moreover Online Phonebook reveals information of 729 drug stores of Karachi. Therefore to correctly represent the population a sample size of 1003 drug store was selected.

A self-made structured closed ended questionnaire was developed for data collection. The questionnaire was designed to collect demographic information regarding drug store, drug storage conditions and practices. The questions were also included to check the regulatory requirements laid down by government authorities including availability and display of valid drug sales license. A team of four data collectors was trained by the principal investigator for a period of one week. A pilot study was done in 51 drug stores to validate and modify the questionnaire and validation of the quality of data. To ensure the quality and consistency of data all the data collectors were asked to meet on weekends with the principle investigator for data sharing. In order to check the transparency of data collection principal investigator randomly used the telephonic verification from the respondents to help overcoming the over or under reporting of the data from the drug stores.

The approval of the study was obtained from the Board of Advanced Studies University of Karachi and formal permission to conduct a survey was obtained by the District Health Office Karachi. An informed consent was obtained from every drug store. All the filled questionnaires were checked and verified by the principal investigator. The data entry was done and descriptive statistics were performed through Stata 11.2. Logistic regression analysis was performed by generating dependent variable “stores meeting licensing requirements” by combining variables i.e. size of store >100sqft, refrigerator present, stores not exposed to sunlight, pharmacist present and valid drug sale license displayed, which are the requirements for the licensing as laid down by government authorities. Another dependent variable was created separating the stores located in the upscale area of Karachi i.e. Defense and Clifton, where majority of the population is high income as compared to rest of areas of Karachi. A univariate analysis was performed to estimate the association of selected factor with the dependent variables and odds ratios, 95% confidence intervals and p-values were calculated to determine likelihood of association.

## RESULTS

In this study, 1003 retail and wholesale drug stores were inspected, with only 3%(n=31) dropout.

There were 99.4%(n=997) stores owned by sole proprietors, 79.5%(n=798) were situated near hospitals/clinics and only 20.4%(n=205) were located near market. Most of the stores 74.9%(n=752) were selling general items along with the drugs and had a mean operating hours of 14h(SD±3.25). There were 19%(n=190) of stores having only one person working on the store, while 55%(n=548) of the stores have the staffing level of 2-3 person. There were only 12%(n=124) stores, in which qualified person (dispensers or pharmacists) were working, out of which 33%(n=41) were pharmacist (Table-I).

**Table-I:** Drug Stores: location, product mix, and operating information.

*Variable/categories*	*F(%age)/Mean(SD)*
Location of drug store	
Market	205(20.4%)
Near clinics & hospitals	798(79.5%)
Sale of general items along with drugs	752(74.9%)
Operating hours of store	14.04(SD±3.25)
Staffing level	
One staff	190(18.9%)
2-3 staff	548(54.6%)
4 staff and above	265(26.4%)
Stores with qualified person.	124(12.3%)
Type of qualified person.1.	
Dispensers	83(66.9%)
Pharmacists.2.	41(33.0%)

1-sign indicate the breakup of medical professionals working on stores, 2-licensing requirement.

In this study only 5.4%(n=54) stores had working air-condition. About 88.6%(n=729) stores inspected, were involved in the sale of vaccines and other refrigerated drugs, out of which 75.6%(n=759) stores had refrigerator for the storage of these products, only 0.2%(n=2) stores had biological refrigerator with proper temperature monitoring. The percentage of stores that were selling refrigerated drugs without proper storage was 11.4%(n=94). Only 11.7%(n=117) stores had the provision of power backup connected to the refrigerators. However 1.4%(n=14) stores were found to demonstrate temperature recording for refrigerators. The washable floor was selected as an indicator of tidiness and 89.8%(n=901) stores were having washable floor, and 40.2%(n=403) stores medicines were protected from direct sunlight. 56%(n=566) stores were found to store drugs directly on the floor (Table-II).

**Table-II:** Storage practices and factors associated as elaborated by Sind drug rules 1979.

*Variable/categories*	*F(%age)*
Stores having Air-conditioning	54(5.4%)
Temperature monitoring devices present and used	14(1.4%)
Refrigerator present.1	759(75.6%)
Biological refrigerator present.2	2(0.2%)
Designated refrigerator for drugs.2	222(22.1%)
Refrigerator with general items.2.	535(53.5%)
Refrigerator on power-backup	117(11.7%)
Stores selling vaccines without refrigerators	94(11.4%)
Floor tidy and washable .1.	901(89.8%)
Drugs protected from sunlight .1.	403(40.2%)
Drugs stored on the floor	566(56.0%)
Storage area >100sqft .1.	815(81.2%)
Out let licensed by Health department	844(84.1%)
Licensed issued to.3.	
Dispenser	379(44.9%)
Pharmacist	465(55.1%)
Type of license.3.	
By way of pharmacy	1(0.1%)
By way of retail sale	803(95.1%)
By way of wholesale	40(4.7%)
Valid License displayed and visible to customers.1,3.	400(47.4%)
License found expired.3.	282(33.4%)

1-licensing requirement, 2-breakup of refrigerator available, 3-breakup of outlet that are licensed

In this study there were 84%(n=844) drug store claiming that have license issued by government authorities. Out of these 844 drug stores, only one drug store had license by way of Pharmacy, 95.14%(n=803) drug stores were having retail sales license and remaining 4.7%(n=40) stores had wholesale license. Only 47%(n=400) stores had visible valid drug sale license displayed to the customers, which is the regulatory requirement by the Ministry of Health, and in 33%(n=282) drug store the drug store license were found expired, 32%(n=321) stores were not able to show the drug sale license. In total 16%(n=159) stores were selling medicines without the drug sales license (Table-II). Based on the above results only 4.1%(n=41) stores were observed to be compliant to the regulatory requirements laid down by government authorities for the sale of drugs.

Univariate analysis was performed to find the likelihood of selected independent variables against the regulatory compliant v/s non-compliant groups identified in the study. Drug stores compliant to the regulatory requirements were more likely to have air-conditioning (OR=3.2,p-value=0.01), temperature monitoring practices (OR=10.2,p-value=0.00), and power backups for refrigerator (OR=2.2,p-value=0.01) showing the statistically significant odd ratio, 95% confidence interval and P-values. On the other hand there was no significant difference in likelihood found between the location of store near hospital/clinic (OR=0.6,p-value=0.3) and practice of storing drugs on the floor (OR=0.5,p-value=0.05) between the regulatory compliant and non-compliant groups (Table-III).

**Table-III:** Univariate analysis for association of independent variables with regulatory compliant and non-compliant group.

*Variable*	*Regulatory compliant stores (n=41), n(%)*	*Regulatory non-compliant stores (n=962), n(%)*	*OR*	*95%CI*	*P-value*
Air-conditioning					
No	35(85.3%)	914(95%)			
Yes	6(14.6%)	48(4.9%)	3.2	1.3-8.1	0.011
T-monitoring practices					
No	37(90.2%)	952(94.9%)			
Yes	4(9.7%)	10(1.0%)	10.2	3.1-34.4	0.000
Refrigerator on power backup					
No	28(68.3%)	798(82.9%)			
Yes	13(31.7%)	164(17.0%)	2.2	1.1-4.4	0.019
Drugs stored on floor					
No	24(58.5%)	413(42.9%)			
Yes	17(41.4%)	549(57.0%)	0.5	0.2-1.0	0.052
Store near hospital/clinics					
No	11(26.8%)	194(20.1%)			
Yes	30(73.1%)	768(79.8%)	0.6	0.3-1.3	0.303

Logistic regression was also performed to assess the association of independent variables with groups made based on the location of stores in different areas of the city. Drug stores located in the upscale areas of Karachi were more likely to have air conditioning (OR=18.2,p-value=0.00), temperature monitoring practices (OR=14.2,p-value=0.00) and power backup (OR=8.2,p-value=0.00) as compared to the stores located in other areas of Karachi. At the same time these stores had less likelihood of drugs being stored on the floor (OR=0.6,p-value=0.00) and exposure to direct sunlight (OR=0.05,p-value=0.00). Furthermore these stores had three times more likelihood of displaying valid drug sales license (OR=3.0,p-value=0.05) as compared to the other group of stores (Table-IV).

**Table-IV:** Univariate analysis for association of independent variables with location of drug stores in different areas of Karachi.

*Variable*	*Stores in upscale areas of Karachi (n=72), n(%)*	*Store in rest of Karachi (n=931), n(%)*	*OR*	*95%CI*	*P-value*
Air-conditioning					
No	46(63.8%)	903(96.9%)			
Yes	26(36.1%)	28(3.0%)	18.2	9.8-33.5	0.000
T-monitoring practices					
No	65(90.2%)	924(99.2%)			
Yes	7(9.7%)	7(0.7%)	14.2	4.8-41.7	0.000
Refrigerator on power backup					
No	30(41.6%)	796(85.4%)			
Yes	42(58.3%)	135(14.5%)	8.2	4.9-13.6	0.000
Drugs stored on floor					
No	66(91.6%)	371(39.8%)			
Yes	6(8.3%)	560(60.1%)	0.6	0.02-0.14	0.000
Store near hospital/clinics					
No	26(36.1%)	179(19.2%)			
Yes	46(63.8%)	752(80.7%)	0.42	0.25-0.69	0.001
Drug exposed to Sunlight					
No	66(91.6%)	337(36.1%)			
Yes	6(8.3%)	594(63.8%)	0.05	0.02-0.12	0.000
Valid License displayed					
No	25(34.7%)	577(61.9%)			
Yes	47(65.2%)	354(38.0%)	3.0	1.85-5.06	0.000

## DISCUSSION

The results shows an alarmingly low number of stores 4.1%(n=41) compliant to the regulatory requirements laid down by government authorities, the number is much less than the study conducted in the capital of Pakistan showing 19.3% compliance.^12^ Out of the five criteria of the licensing requirements that were assessed the lowest was the sales of medicine under the supervision of pharmacist, which is also observed in the other studies of the region.^11^,^12^ Drug outlets being the first point of contact for the patients and prevailing practice of self-medication coupled with such low regulatory compliance makes the safe use of drug even more challenging in the largest urban population of the country.^3^,^7^,^9^ Only 19% of the stores show the presence of drug sellers with degree in medicine/pharmacy or having dispenser diploma which means the majority of the drug sales are carried out by people without any formal education about medicine. The findings are consistent with the other studies ranging from 12-22% of drug sellers who are qualified or trained.^11^,^12^

Only 25% of the stores were selling drugs exclusively, majority of the stores were also selling general items, food items, cosmetics etc, making the drug stores a commercial facility rather professional health care delivery service. More than one third of the stores were located near the hospitals and clinic, and majority operating for 11-17 hours showing they serve a large number of populations and are viable business entities. Similar business-like environment at pharmacies is also reported from India and other developing countries.^14^

Almost 95% of the stores didn’t have the provision of air conditioning in the store, and almost none of them have temperature monitoring practices in place, further more half of the stores inspected had a design where by drugs are exposed directly to sunlight. These compromised practices of storage in a hot and humid city like Karachi, makes the quality of the products questionable. A previous study does suggest the exposure to high temperature cause the medical products to lose their efficacy^15^, and the study conducted in Pakistan show the similar percentage of stores with the air conditioning, while more of the stores were recording temperatures.^12^

About 19% of drug stores were having covered area of <100 sq ft, which violates one of the basic requirement of licensing, suggesting that at the time of issuance of license all the parameters of storage are not checked properly, and furthermore this is also not verified during periodic inspections of the facilities by the regulators. Scarcity of space leads huge number of drug stores to keep drugs directly on the floor which makes it difficult to keep the store clean and tidy. Another parameter for tidiness i.e. “was the floor washable” was also reviewed revealing the similar findings of 90% store having smooth washable floor as previous study,^12^ while the remaining 10% of the stores were carpeted.

The storage of temperature sensitive drugs requiring refrigeration (temperature range between 2-8C) was grossly compromised. There were 11.4% of the stores selling refrigerated drugs (e.g. vaccines, insulins etc) without proper refrigerator which does pose serious implications on the efficacy of the vaccines impacting the portion of the vaccination program covered by private drug sellers. Almost all the stores were using domestic refrigerators and main commodity stored was general items i.e. soft-drinks etc, making it inappropriate for the proper storage of vaccines and other refrigerated drugs. Very few of the stores had the backup power supply attached to the refrigerator, which is a serious concern because of high frequency of power cuts in the city due to energy crisis. These practices reveal that mostly people are either unaware or ignorant towards of the storage conditions of the vaccines and other biologicals and treat them general commodities which can be analyzed in future studies. Similar kinds of finding were observed in previous studies from Pakistan and India. In a similar study from Karachi more than half of pharmacies were keeping vaccines in compromised storage conditions.^11^,^12^,^16^

It is observed that almost half of the stores had the license issued in the name of pharmacist, and in only 41 stores pharmacist was physically present, it does suggest that the pharmacist provide their degrees to the drug stores mostly for the issuance of license to cover them legally while physically they don’t work on the outlet or might come periodically. Therefore most of the drug sale is carried out under supervision of unqualified drug seller’s rather qualified pharmacists. It seems that pharmacists only provide them the legal cover to operate the drug sales. Similar study conducted in Sri Lanka demonstrates qualified pharmacists usually own chain of pharmacies, and visit them periodically.^17^

A drug sales license once issued is valid for two years and facility has to be inspected for the renewal of license. Surprisingly one third of the stores that were licensed by provincial government of health were having expired licensed. Most of the drugs sellers mentioned that they had applied for the renewal of the license; this shows a poor control and inspection practices by the regulator, and suggests an almost non existing regulatory mechanism for the re-inspection of licensed facilities.

## CONCLUSION

The regulatory compliance of majority of the drug stores operated privately in different areas of Karachi is below standard. Only a few drugs stores have mechanism to protect the drugs from extreme storage temperature, sunlight and had the provision of refrigeration. Very few of the stores carry out drug sales under the supervision of qualified pharmacist. There are stores operating without drug sales license and selling refrigerated drugs without having proper facilities for the storage. Therefore majority of the drug stores in Karachi can be classified as commercial facility selling drugs as one of the commodity in there store, rather than professional community pharmacy services. There were significant difference observed between the storage practices and regulatory compliances of the stores with respect to the location of the store when categorized between high income and middle/low income areas of Karachi. The alarmingly low compliance of the drug stores to the regulatory requirements observed in this study does suggest serious lapse in the regulatory framework involving every step from the issuance of license till the monitoring of stores, and does conclude that necessary steps to be taken by regulators and other stakeholders to improve the drug sales practices.
